# Supplementary material for: Characterization of diffusing sub-10 nm nano-objects using single anti-resonant element optical fibers
Source: Nat Commun. 2023 Jun 5;14:3247. doi: 10.1038/s41467-023-39021-3 (PMC10241938; doi:10.1038/s41467-023-39021-3)
Supplement: Supplementary file 1 — Supplementary Information [file 41467_2023_39021_MOESM1_ESM.pdf]

Supplementary Information

Characterization of diffusing sub-10 nm nano-objects using single anti-resonant element optical fibers

Torsten Wieduwilt, Ronny Förster, Mona Nissen, Jens Kobelke and Markus A. Schmidt

Content

1. SI 1: Design of the chip 2
2. SI 2: Diameter analysis 3
3. SI 3: Background 4
4. SI 4: Exposure time and framerate 5
5. SI 5: Summary of the results of measurements 6
6. SI 6: Impact of photon drift 8
7. SI 7: Impact of nanoparticle heating 9
8. SI 8: Characterization of 7 nm gold nanoparticles 11
9. SI 9: Comparison of DLS and FaNTA 11
10. SI 10: Stability of light transmission 13
11. SI 11: Single nanoparticle reliability of measurement system and data analysis 14
12. SI 12: Wall thickness dependence 15
13. SI 13: Optimization of modal attenuation 16
14. SI 14: Measurements of modal attenuation 17
15. SI 15: Resistance factor 18
16. SI 16: Impact of confinement on MSD analysis 20
17. SI 17: Influence of the trajectory length on the accuracy of ensemble statistics 22
18. SI 18: Simulation of particle diffusion for large lag times 23
19. SI 19: Optimization of light incoupling 24
20. SI 20: Description of z-score filtering 25
21. List of mathematical symbols 27
22. Bibliography 29

1. SI 1: Design of the chip

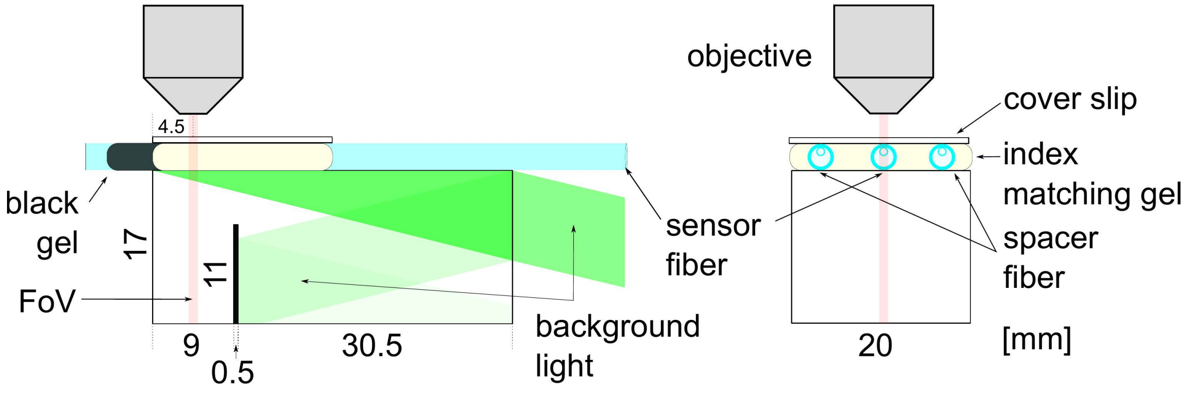

Fig. S-1: Design of the chip along the fiber (left) and in cross section (right)

The key for measuring small particles is to reduce the background signal as much as possible. It is important to note, that even the slightest reflection of the illumination into the objective is very intense compared to the elastically scattered light of nanoparticles. Even rays that have been reflected several times must not enter the lens or the field of view. This is done by avoiding background in the first place and directing unavoidable background away from the field of view. In addition, everything should have the same refractive index of fused silica, to prevent reflections in unwanted directions. To fulfill these requirements, we have developed our own chip as a specimen holder (see Fig. S-1).

The first step is to minimize the amount of light which is not in the measurement channel. We optimized the efficiency of the butt coupling, by using a delivery fiber with an output mode as close to the fundamental mode of the SEF as possible, which has a large diameter (d_c = 17 µm) and very low NA (0.024). This is done by a single mode fiber (S405-XP, Thorlabs) with a customized fiber (single mode; NA = 0.05; core = 7µm) spliced to its end. Nevertheless, some light will be outside the ARE inside the glass. Thus, we apply a drop of index matching gel (G608N3, Thorlabs) on the SEF after the butt coupling. We added some soot to the gel to absorb the outcoupled light. This step is required for gold particles of 20 nm diameter and below.

The SEF is covered by a coverslip made of fused silica (*d* = 400 µm). The coverslip is horizontally aligned by two empty SEF on each side of the filled SEF. A glycerin-water-mixture $(n(\lambda = 532nm, 20°C) = 1.46)$ is used as an index matching gel under the

coverslip. The gel has a high viscosity and a convenient contact angle, so that it flows quickly under the coverslip without draining off.

Thus, the scattered light from the nanoparticles leaves the fiber without significant aberrations. In addition, the index matching gel increases the losses of the higher modes so that this light leaves the fiber.

To get this light away from the field of view, the SEF is placed on a customized fused silica block, which the outcoupled light can enter without any reflection or refraction and is visible as a cone around the SEF. Although most of the light will leave the glass block, some will be reflected by the glass-air-surface. To avoid stray light into the field-of-view, the glass block is polished on every side, so that the reflected light beam goes further down the block. But it cannot reach the field-of-view, because a slit is sawed into the block and filled by cardboard.

The objective is placed around 4.5 mm behind the edge of the coverslip. This is the position with the lowest background.

The output of the SEF is sealed by the self-made index matching gel. The fiber end needs to be closed so that no flow occurs inside the channel caused by evaporation of the water. Using the gel is convenient, because it allows to image the fiber mode by attaching a coverslip to it.

2. SI 2: Diameter analysis

The diameter determination procedure employs mean squared displacement (MSD) analysis for each particle individually and is defined by:

Theory: $$\mathrm{MSD}(i\Delta t) := < (x(t) - x(t + i\Delta t))^2 > = 2 \cdot i\Delta t \cdot D \tag{1}$$

Experiment: $$\mathrm{MSD}(i\Delta t) \approx \frac{\sum_{j=0}^{N_\mathrm{f}-i} \left(x_j - x_{j+i}\right)^2}{N_\mathrm{f}-1} = 2 \cdot i\Delta t \cdot D + (v \cdot i\Delta t)^2 + \sigma^2 \tag{2}$$

with lag-time Δt, lag-frame *i*, trajectory length N_f, diffusion coefficient *D*, drift velocity v and localization accuracy σ^2 (< > is the expectation value operator). The expectation value is approximated by averaging over all measured displacements given by the measured trajectory. The drift velocity v should be 0 at any time in the experiment. Then, the diffusion coefficient can be retrieved as the slope of the MSD curve. The ideal number of considered lag-frames *i* is set by the experimental parameters [1].

The NP diameter is retrieved by the Stokes-Einstein equation considering the hindrance factor, which is yet close to 1 [2]. Note that the more frames N_f a continuous trajectory consist of, i.e., a NP is tracked, the more accurate is the approximation in Eq (2), thus leading to a lower statistical error σ_D. Furthermore, the error is influenced by the localization accuracy and the particles motion blur, leading to a total minimum relative error of [1,3]:

$$\frac{\sigma_\mathrm{D}}{D} \gtrsim \sqrt{\frac{2}{N_\mathrm{f}-1}} \tag{3}$$

Eq (2) and (3) represent the key advantages of NTA: If a particle can be tracked, its individual diameter can be retrieved and the precision can be tuned by the number of frames N_f the movement is captured. The CV is a standardized measure to quantify the precision of a statistically limited measurement process such as NTA.

3. SI 3: Background

Fig. S-2 shows the background signal of the ARE inside the SEF, by filling the fiber with ultra-pure water (a) and ethanol (b) only (NP-free liquid). As the experimental conditions were the same in both cases, a quantitative comparison of the amount of scattered light is possible by summing up the intensity counts in the area of the microchannel (yellow rectangular in Fig. S-2, accumulated counts are given on the left-handed side). The ratio of the resulting values is 2.9, which agrees well with the ratio of the Rayleigh ratios reported in literature (Rayleigh ratios: $R_\mathrm{ethanol} = 3.92 \cdot 10^6 \mathrm{cm}^{-1}$; $R_\mathrm{water} = 1.34 \cdot 10^6 \mathrm{cm}^{-1}$, $\frac{R_\mathrm{ethanol}}{R_\mathrm{water}} = 2.92$) [4] and proving that the background signal truly originates from the liquid and not from fiber losses or experimental inaccuracies.

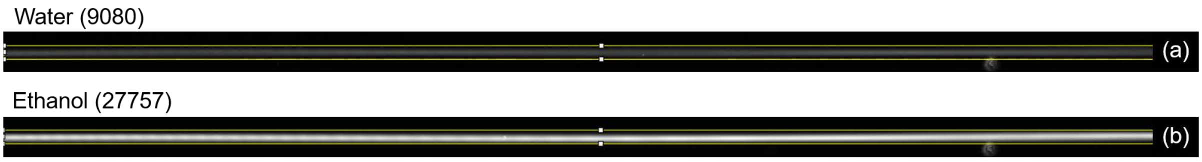

Fig. S-2: Image of the scattered background light acquired by filling the ARE fiber with (a) water and (b) ethanol under identical conditions. No nanoparticles are involved in this experiment. The yellow rectangle indicates the region of the ARE in which the intensity counts were summed up (the respective value is given on the left-handed side of the images).

4. SI 4: Exposure time and framerate

Exposure time and framerate should be as large as possible, in order to get maximum signal and sample points of the trajectory N_f, minimizing the statistical error in the MSD-analysis [1]. However, both parameters oppose each other. For the 9 nm AuNP, we performed the FaNTA for three different exposure times and framerate, resulting in comparable hydrodynamic diameters (650 fps: μ = 16.2 nm, 450 fps: μ = 16.1 nm and 250 fps: μ = 15.9 nm, see Fig S-3), verifying that the retrieved diameter by FaNTA is independent of exposure time and framerate. While the 9 nm AuNPs have not been visible for exposure times below 1 ms, the bright 50 nm particles can be imaged with an exposure time down to 150 µs, allowing a framerate in the KHz-regime. For a fair comparison of all NPs, we compare datasets with identical framerate (450 fps).

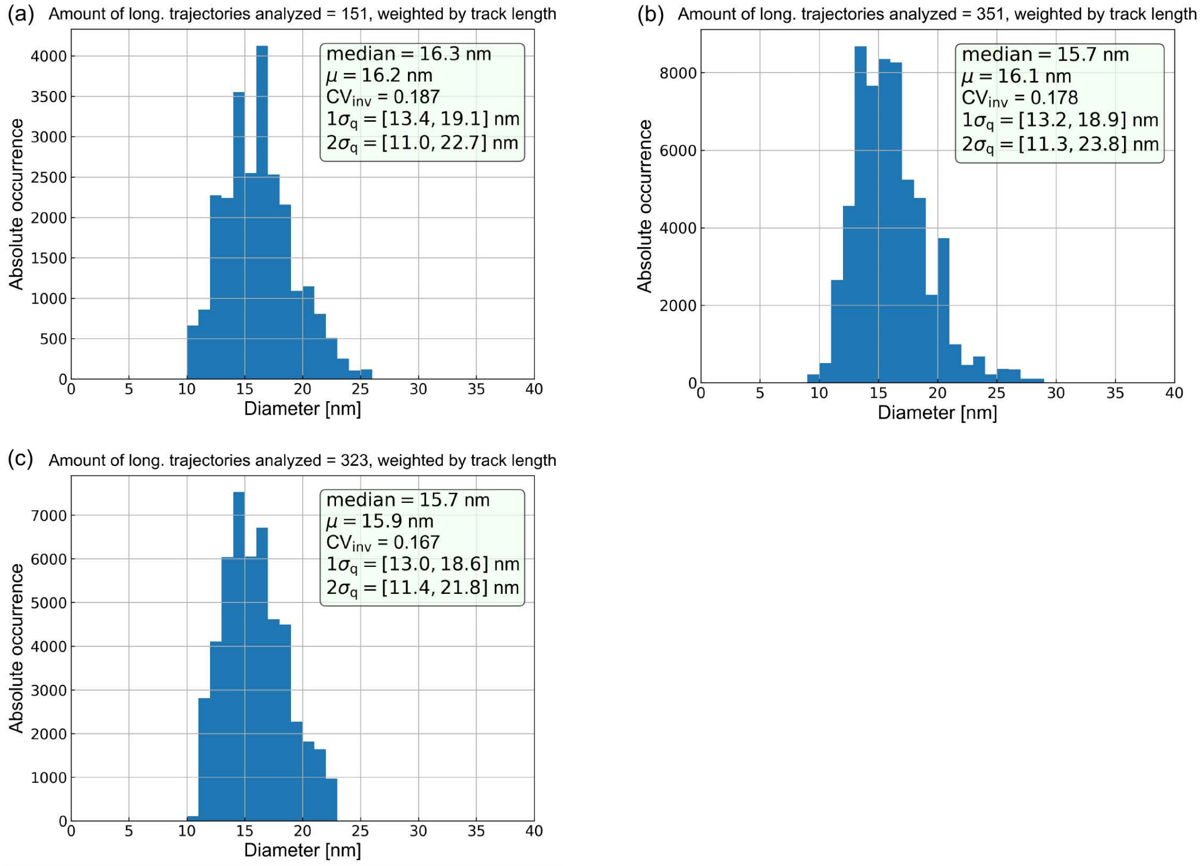

Fig. S-3: Retrieved hydrodynamic diameter of 9 nm AuNP for different exposure times and framerates: a) 1 ms @ 650 fps; b) 1.9 ms @ 450 fps and c) 3.7 ms @ 250fps.

5. SI 5: Summary of the results of measurements

	Unit		9 nm	20 nm	30 nm	50 nm	50 nm	25 nm
Material			gold				polystyrene	DNA
Product Number			AUXU10	AUXU20	AUXU30	AUXU50	3050A	
Lot Number			JSF0095	JLF0261B	JSF1024	PSK0163	193396	
Vendor			nanoComposix				ThermoFisher	
$d_{\text{TEM, m}}$	nm	Manufacturer	8.8 ± 0.4	20.4 ± 0.5	28 ± 0.9	50.3 ± 2.3	51 ± 3	25
CV	%		4.4	2.5	3.1	4.5	16.5	
$d_{\text{DLS, a}}$	nm		19	28	36	60	-	
Particle Surface			PEG12; carboxylic acid					
d_{DLS} (Zetasizer Nano ZS and Ultra; Malvern)	nm		17.9	28.1	35.0 ± 0.5	57.4 ± 0.0	64.9 ± 1.7	
$d_{\text{FaNTA-trans}}$	nm	Measured	17.4 ± 0.3	27.5 ± 0.6	35.7 ± 0.5	62.0 ± 1.2	64.7 ± 1.1	
$d_{\text{FaNTA-long}}$	nm		16.1 ± 0.1	26.5 ± 0.6	33.9 ± 0.7	59.2 ± 1.6	62.8 ± 0.9	
σ^2_{abs}	nm^2		34	44	1193	7 137	$3.9 \cdot 10^{-17}$	0
σ^2_{scat}	nm^2		0.026	4.3	29	987	2.2	0.030
σ^2_{ext}	nm^2	Theory	34	49	1222	8 124	2.2	0.030
$\sigma^2_{\text{abs}}/\sigma^2_{\text{scat}}$			1 315	10	41	7	$1.7 \cdot 10^{-12}$	0
$P^{(\text{heat})}_{\text{max,det.pos.}}$	mW		417	321	12	~2 [measured]	$3.66 \cdot 10^{20}$	∞

$P_{\text{max,det.pos.}}^{\text{(photon pressure)}}$	mW		2 850	2 000	80	12 [measured]	44 700	$3.3\cdot 10^{6}$

Tab. S-1: Summary of different measured and theoretical values of the investigated NPs. Cross-sections calculated by https://nanocomposix.com/pages/mie-theory-calculator.

The calculated values for $P_{\text{max,det.pos.}}^{\text{(heat)}}$and $P_{\text{max,det.pos.}}^{\text{(photon pressure)}}$refer to the laser power limits determined experimentally on the 50 nm AuNp. For the calculation of $P_{\text{max,det.pos.}}^{\text{(heat)}}$ and $P_{\text{max,det.pos.}}^{\text{(photon pressure)}}$, the ratio of the absorption cross-sections and the extinction cross-sections was used. The following refractive indices at the operation wavelength (λ = 532 nm) were used for the calculations of both cross-sections: n_{water}=1.3354, $n_{\text{polystyrene}}$=1.598 and n_{DNA}=1.59.

6. SI 6: Impact of photon drift

Fig. S-4 shows the influence of laser power on particle drift using the example of 50 nm AuNP. The drift is estimated as the average movement of all particles per frame. It can be seen that particles drift only along the fiber direction (longitudinal; red curve in Fig. S-4), and that the drift speed is a linear function of the laser power. The maximum laser power a measurement should not exceed can be calculated:

Under ideal condition only two MSD values ($i = 1, 2$) are required to retrieve the diffusion coefficient. In case of constant drift velocity v we retrieve the diffusion coefficient to:

$$D_v = \frac{\mathrm{MSD}\,(2\cdot\Delta t) - \mathrm{MSD}\,(1\cdot\Delta t)}{2\cdot\Delta t} \tag{4}$$

$$= \frac{2D\cdot(2\cdot\Delta t) + (v\cdot 2\cdot\Delta t)^2 - \left(2D\cdot(1\cdot\Delta t) + (v\cdot 1\cdot\Delta t)^2\right)}{2\cdot\Delta t} \tag{5}$$

$$= \frac{2D\cdot\Delta t + 3\cdot(v\cdot\Delta t)^2}{2\cdot\Delta t} = D + \frac{3\cdot(v\cdot\Delta t)^2}{2\cdot\Delta t} \tag{6}$$

The ideal measurement is drift free ($v = 0$), leading to: $D_{v=0} = D$.

The rel. error ε of D induced by the drift can be expressed by the drift induced displacement $\Delta x_{\mathrm{drift}} = v \cdot \Delta t$ and the expected brownian motion $< \Delta x_{\mathrm{brown}} >= \sqrt{2 \cdot \Delta t \cdot D}$ between two frames:

$$\varepsilon = \frac{D_v - D}{D} = \frac{\frac{3\cdot(v\cdot\Delta t)^2}{2\cdot\Delta t}}{D} = \frac{3\cdot(v\cdot\Delta t)^2}{2\cdot\Delta t\cdot D} = \frac{3\cdot\Delta x_{\mathrm{drift}}^2}{\Delta x_{\mathrm{brown}}^2} \tag{7}$$

The rel. error is a quadratic function of the velocity, which depends linearly on the photon pressure and thus the laser power. Fig. S-4 (blue curve) shows the measured rel. error over the laser power and confirms the quadratic dependency. The laser power corresponds to the value measured at the outlet of the ARE fiber (*L*= 14 cm). The power at the observation point (tracking detection point) is higher by a factor of ~2.1. Assuming that the rel. error should not exceed 1 %, the maximum value can be obtained out of the parabolic fit function:

$$\varepsilon_{\mathrm{max}} = 1\% = A + B \cdot P + C \cdot P^2 \tag{8}$$

$$\rightarrow P_{\mathrm{max-out}}^{(\mathrm{photon\ pressure})} = 6\ \mathrm{mW} \tag{9}$$

In relation to the laser power at the tracking detection point, this corresponds to a value of ~ 12.5 mW. The measured hydrodynamic diameters $d_{FaNTA\text{-}trans}$ and $d_{FaNTA\text{-}long}$ (see Tab. S-1) correspond to the mean value of all measurements below the power threshold at the tracking detection point (below ~12.5 mW in case of the 50 nm AuNp). With the help of the theoretical known scattering and absorption cross section, it is possible to transfer $P_{\text{max}-\text{out}}^{(\text{photon pressure})}$ to any other particle [see Tab. S-1 in SI 5], demonstrating that none of our measurements suffers from photon pressure.

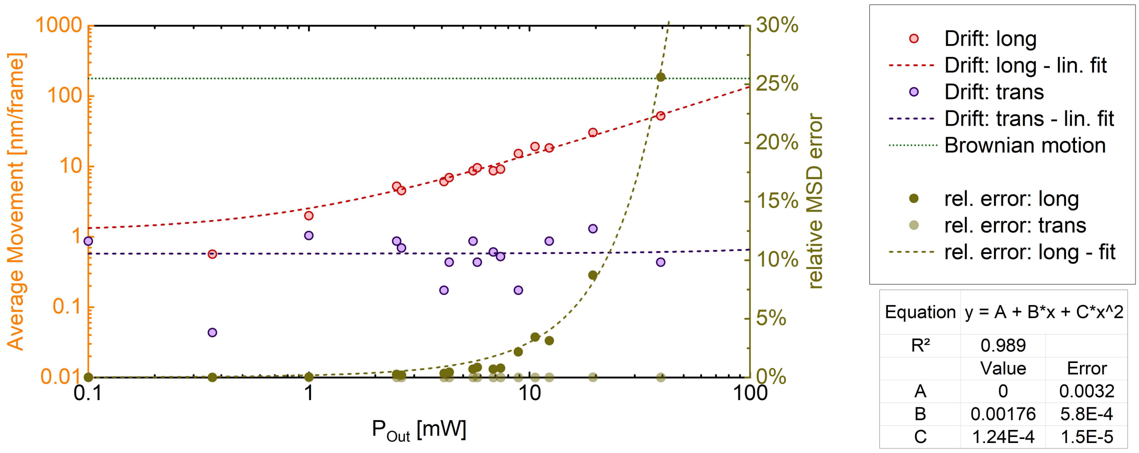

Equation	y = A + B*x + C*x^2	
R²	0.989	
	Value	Error
A	0	0.0032
B	0.00176	5.8E-4
C	1.24E-4	1.5E-5

Fig. S-4: Average movement of all 50 nm AuNP over the laser power. One frame corresponds to 2.22 ms.

7. SI 7: Impact of nanoparticle heating

Figure S-5 shows that the retrieved diameter decreases with the used laser power. This is not an effect of the photon pressure only, because diameter decreases in the transversal direction, too, which is not affected by photon pressure. The reason is most likely of thermal nature. The NP are heated up by the absorbed photons. The dissipating heat increases the temperature of the surrounding water leading to a reduction of its viscosity and a higher diffusion coefficient of the particles. If the viscosity change is not corrected, the particles retrieved diameter is too small, as it happens in Fig S-5. As can be seen in Fig. S-5, the diameter is reduced for laser powers greater than ~3 mW (laser power measured at the output of the ARE fiber; corresponds to a laser power at the tracking detection point of ~6 mW).

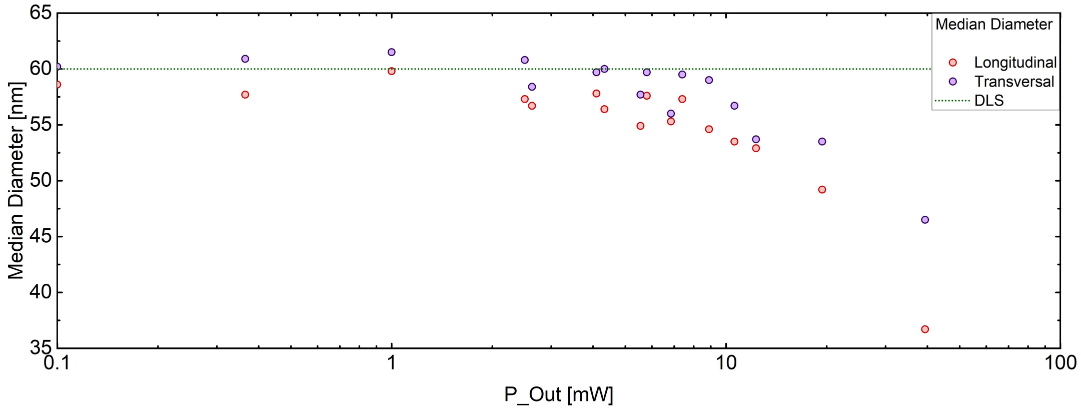

Fig. S-5: Average median diameter on example of 50 nm AuNP over used laser power.

Considering that the particles diameter is not affected by the laser power, the viscosity of the water can be retrieved by the Stokes-Einstein-Equation, leading to the temperature of the water by using a lookup-table (Fig S-6) [5]. Note that the longitudinal evaluation suffers from additional motion due to the photon pressure, leading to an overestimation of the diffusion coefficient. Thus the viscosity is estimated too low leading to an overestimated temperature. The data is fitted by a linear function, because the absorbed energy of the particle is proportional to the incoming laser power for small temperature changes. The determined systematic error of unnoticed particle heating in the retrieved diameter is 3 %/ K [3]. Again, we limit the systematic error to 1 %, which corresponds to a maximum temperature rise of 0.33 K. The fit function in Fig. S-6, leads to a maximum Power $P_{\mathrm{max}}^{(\mathrm{heat})}$ of 1 mW (corresponds to ~2 mW at the tracking detection point) for the 50 nm AuNP.

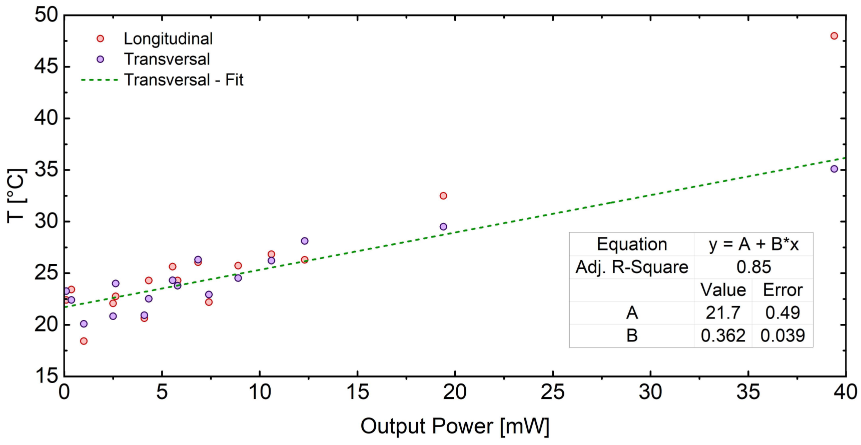

Fig. S-6: Estimated water temperature over used laser power.

With the help of the theoretical known scattering and absorption cross-section, it is possible to transfer $P_{\max}^{(\text{heat})}$ to any other particle [see Tab. S-1 in SI 5], demonstrating that none of our measurements suffers from thermal problems.

8. SI 8: Characterization of 7 nm gold nanoparticles

We would have liked to investigate whether we see anything beyond the demonstrated 9 nm AuNP. However, there are no stable and ultra-uniform AuNP available for 7 or 8 nm. The best specimen we could get are 7 nm AuNP with a CV of 10 % (AUCB7, nanoComposix). The specimen contains a significant amount of NP larger than 9 nm. However, these particles are not distinguishable from the intended 7 nm NP by our method. Thus this specimen is not suitable to verify that we can see a 7 nm NP. In addition, we see agglomerations which did not occur with the other presented NP coming from the same manufacturer. Agglomerations can be found even after filtering the specimen by a 20 nm filter, indicating that the agglomerates reform quickly after the filter and cannot be avoided. In addition, the 20 nm filter blocks a significant amount of particles.

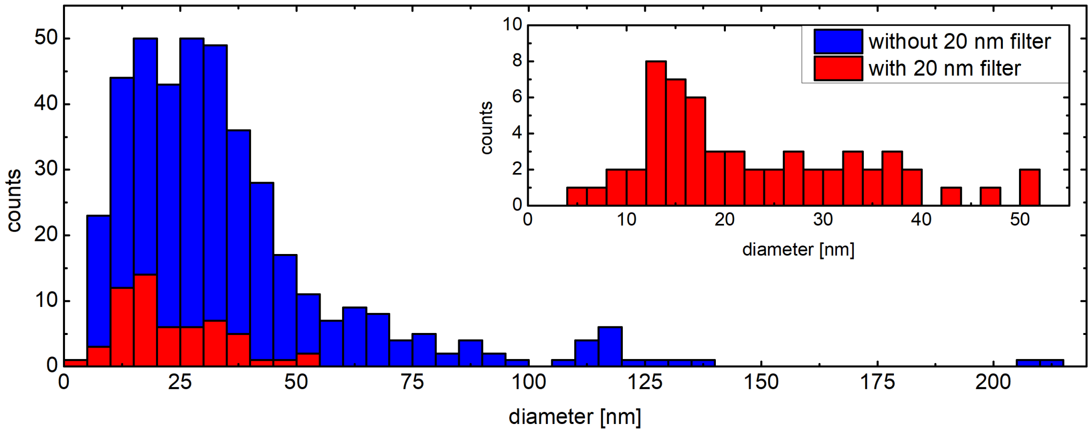

Fig. S-7: Retrieved diameter of 7 nm AuNP by FaNTA shows many agglomerations besides the expected peak around 15 nm.

9. SI 9: Comparison of DLS and FaNTA

An important distinction between dynamic light scattering (DLS) and NTA is that in DLS the temporal behavior of the scattering intensity is evaluated. A consequence of this approach is that the determined hydrodynamic diameter of the relevant NPs

depends on the concentration of the solution investigated. Thus, a concentration series must be performed in order to determine the correct diameter. To demonstrate this behavior in the context of the study discussed here, DLS-related experiments using on our in-house DLS-instrument (Zetasizer Nano ZS and Ultra; Malvern Panalytical) were performed by successively diluting a highly concentrated solution of gold NP (physical diameter 50nm) and characterizing it in the Zetasizer (Fig. S-8 (a)). In accordance with the discussion above, a very strong concentration dependence was found. For comparison, FaNTA experiments were performed with a specific solution (diluted with 0.1 % TWEEN20, concentration $10^{-8}\mathrm{NP/\,mL}$), which revealed an average diameter of 64.3 nm (Fig. S-8 (b)). This clearly shows that diameter determination in DLS always requires a concentration series, which is not necessary when using FaNTA.

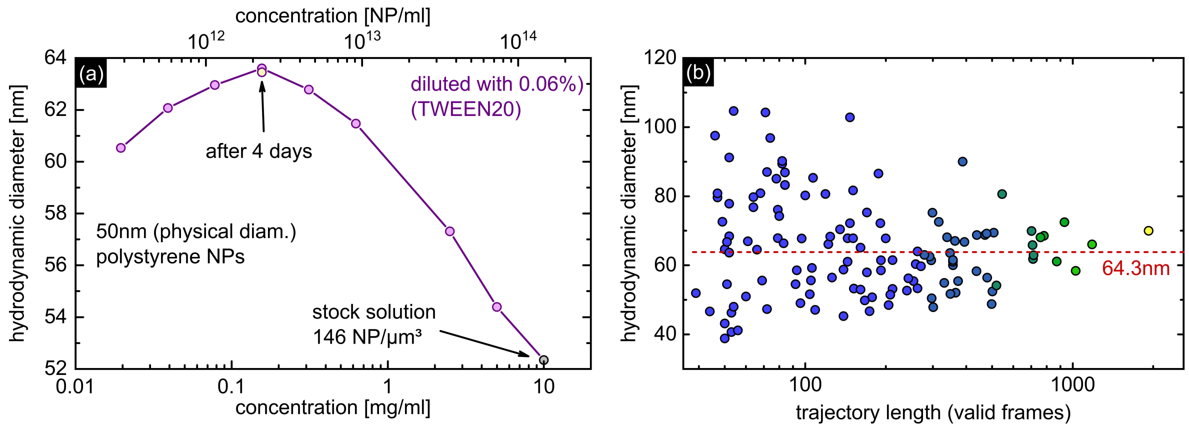

Fig. S-8: Comparison of DLS and FaNTA regarding diameter determination of NPs. (a) Concentration series measured by using our in-house DLS machine showing the concentration dependence of the determined hydrodynamic diameter being intrinsic to DLS (physical diameter 50 nm) (top axis: number concentration. Bottom axis: mass concentration (density)). The dots refer to the mean diameter of the respective DLS measurement, where each measurement was realized by multiple dilution of the initial stock solution (gray dot). The light yellow dot shows a reference measurement at the concentration yielding the largest hydrodynamic diameter after four days, confirming the reproducibility of the measurement principle. (b) Results of the corresponding FaNTA measurements. Shown is the diameter distribution as a function of trajectory length. The color bar

refers to the respective trajectory. The resulting mean hydrodynamic diameter is 64.3 nm (horizontal red dashed line).

These results clearly show that certain assumptions must be generally considered in case a DLS-based analysis is used, whereby the results cannot be interpreted unambiguously. In contrast, FaNTA requires the intensity only for NP localization and not for the actual determination of the hydrodynamic diameter, since FaNTA solely analyzes the trajectory of the individual NPs. Thus, no concentration dependence of the diameter to be determined is present for FaNTA, which clearly reveals that FaNTA is fundamentally more independent of any initial assumptions.

10. SI 10: Stability of light transmission

An example of the time evolution of the power transmitted through the water-filled microchannel (normalized to the average value $P_{\mathrm{n}} = P/P_{\mathrm{ave}}$) over a period of 30 min is shown in Fig. S-9. Experimentally, the light originating from the microchannel at the fiber output was imaged onto a Si-photodetector by means of a lens and a pinhole. A negligible variation in output power over this period is observed (standard deviation $\sigma_{\mathrm{P}} = 0.0045$), demonstrating the stability of intensity at the location of the NPs over a time period that is substantially longer than that of the experiments discussed here. Note that the power variations are largely caused by the butt coupling between the delivery fiber and the ARE-fiber, which can be principally improved by an optimized design of the sample mount.

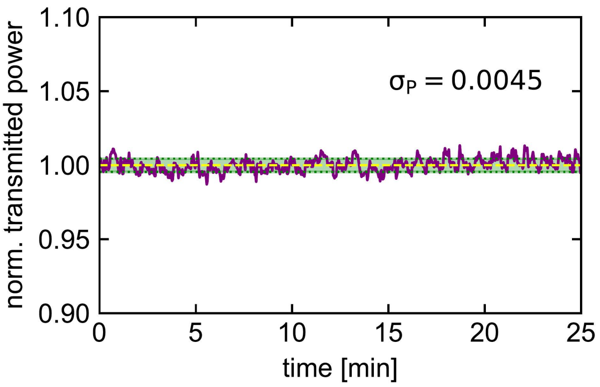

Fig. S-9: Normalized transmitted power at the output of the water-filled ARE as a function of time over a period of 30 minutes. The data is normalized to the mean value of the output power (yellow dashed line). The green area represents the range corresponding to a width of the first standard deviation

($P_n = P_{ave} \pm \sigma_P$), i.e., the range in which 68.2 % of the power values fall. The standard deviation is $\sigma_P = 0.0045$.

11. SI 11: Single nanoparticle reliability of measurement system and data analysis

An important parameter for revealing the reliability is the coefficient of variation (CV), which is basically defined as the ratio of standard deviation and mean value. In the case of NTA, the CV is generally defined by $\mathrm{CV_M} = \sqrt{\mathrm{CV_E^2} + \mathrm{CV_S^2}}$ with the measured CV$\mathrm{CV_M}$ and the CVs of the NP ensemble $\mathrm{CV_E}$ and the system $\mathrm{CV_S}$. Thus, it can be inferred that the parameter $\mathrm{CV_S}$ can serve as an indicator for the reliability of the measurement system and the associated data analysis. In order to eliminate the influence of the size distribution of an NP ensemble, i.e. the influence of $\mathrm{CV_E}$, a series of measurements was carried out in which the same NP ensemble was successively characterized 10 times. Thus, $\mathrm{CV_E} = 0$ and the measured CV characterizes the reliability of the system ($\mathrm{CV_M} = \mathrm{CV_S}$). The resulting values for the determined mean diameter with the corresponding CV including the measurement conditions used are summarized in the following table (Tab. S-2).

Tab S-2: Results of reliability study, using 10 individual sequential measurements of the same gold nanoparticle ensemble (nanoComposix) within a total period of $T = 15$ min. The measurement conditions are as follows: frame rate $\mathrm{FR} = 450$ fps, image size: (80×4096) pixel $(0.028\ \mathrm{mm} \times 1.42\ \mathrm{mm})$, exposure time $\tau = 1.9$ ms, time of each measurement: $t_m = 20$ s (9000 images), time between measurements $t_m \approx 1.5$ min.

measurement no.	median [nm]	average [nm]	CV
1	54.8	53.1	0.059
2	52.5	52.3	0.036
3	53.3	53.1	0.048
4	52.8	53	0.056
5	51.7	52.8	0.095
6	51.8	52.1	0.063
7	52.6	52.3	0.06

8	53.6	53.7	0.058
9	50	51.5	0.066
10	54.1	54.3	0.026

The data analysis yields a hydrodynamic diameter averaged over all ten measurements of $d_{\mathrm{all}} = 52.82\ \mathrm{nm}$ with a standard deviation of $\sigma_{\mathrm{all}} = 0.77\ \mathrm{nm}$, resulting in a system-related CV of $\mathrm{CV_S} = 0.01$. This low value clearly confirms the excellent single NP reliability, i.e. the reliability of the combination of measurement system and appropriate data analysis and its potential for the characterization of nano-objects in general.

12. SI 12: Wall thickness dependence

Crucial to the optical behavior of the SEF is the light guiding mechanism within the ARE, which in the case of the fiber used here is the anti-resonant effect. Since this effect relies on interference, transmission bands that are limited by strong resonances emerge in the spectral distribution of the transmitted power. To spectrally locate the operation wavelength ($\lambda = 532\ \mathrm{nm}$) into one of the transmission bands (i.e. low-loss region), an appropriate wall thickness of the silica-based ARE was chosen during fiber fabrication ($w = 730\ \mathrm{nm}$). To emphasize that the wall thickness dependence is essential, we have calculated the distribution of the modal attenuation of the fundamental mode in an annulus having the same diameter as the ARE used in the experiments at the operation wavelength for the air (red) and water (blue) cases (Fig. S-10):

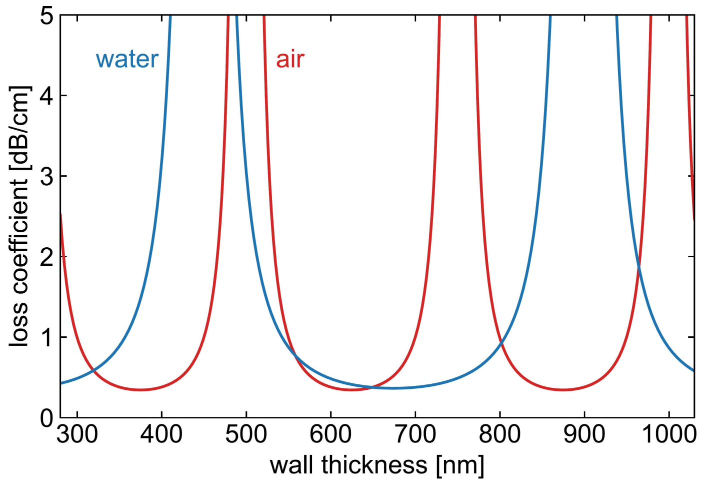

Fig. S-10: Simulation of modal loss of the fundamental core mode of an annulus waveguides (material sequence: liquid/silica/liquid) as a function of

wall thickness at the operation wavelength ($\lambda = 532$ nm). The core diameter was chosen to match the diameter of the ARE used in the experiment ($17\ \mu m$). The two colors refer to water (blue) and air (red).

13. SI 13: Optimization of modal attenuation

One crucial aspect that needs to be considered in case anti-resonant waveguides are employed are the modal losses, which need to be sufficiently low to ensure a constant light intensity longitudinally over the entire field-of-view. The main loss distribution in the present type of anti-resonant waveguide is the sintering point between ARE and cladding, which intrinsically has low reflectivity due to the absence of interference between two interfaces. To illustrate this effect, the influence of the geometry of the ARE-jacket junction was simulated via FEM by modeling the modal losses of the core mode at the operation wavelength ($\lambda = 532$ nm, water-filled case). Note that the azimuthal asymmetry of the ARE cross-section results in birefringence, which removes the degeneracy of the fundamental mode of the ideal cylindrical structure, leading to modal splitting of the fundamental mode. As shown in Fig. S-11(a), the modal loss increases as the width of the junction is increased. To find a compromise between losses and size of the contact point, a junction width of $w = 2\ \mu m$ (Fig. S-11(d)) was chosen in the experiments reported here. The corresponding simulations showed losses of $\gamma_{sim} = 0.4$ dB/cm, yielding a reduction of the power transmission over the length of the field-of-view ($L = 1.4$ mm) of $\Delta T_p = 1.3$ %. This reduction is practically negligible and thus a constant illumination of the NP across the entire measurement area can be assumed.

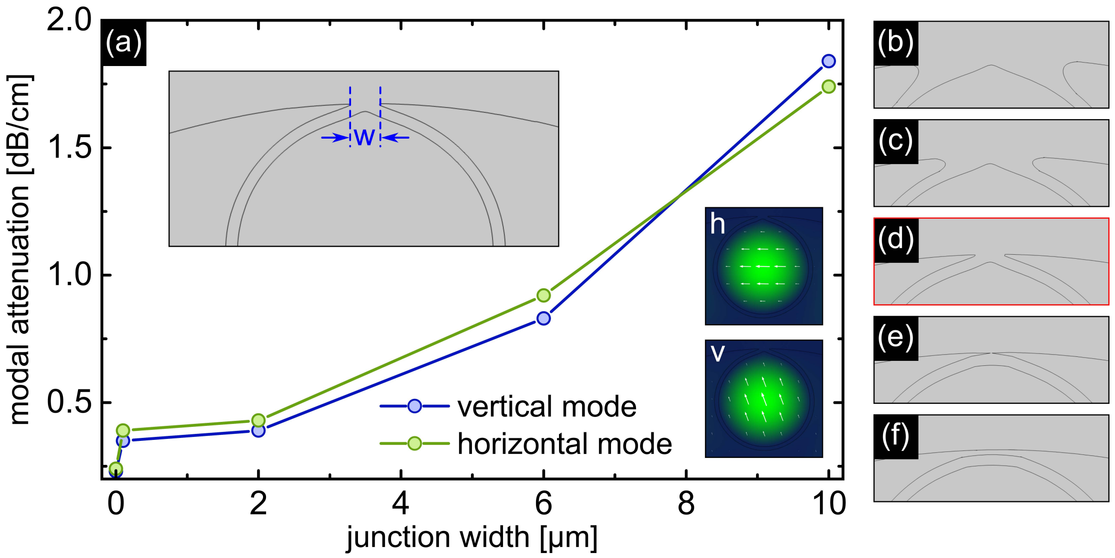

Fig. S-11: Impact of the junction between the ARE and the cladding on the modal attention of the fundamental ARE modes at the operation wavelength ($\lambda = 532\ \mathrm{nm}$, water-filled case). (a) Modal attenuation as a function of the width of the junction of the two fundamental modes. The inset on the top left defines the width parameter w. The intensity distributions of the two modes (h: horizontal, v: vertical) for $w = 2\ \mu\mathrm{m}$ are shown in the lower right insets (white arrows: direction of electric field at a fixed point of time). The different configurations simulated are shown in images on the right ((b) $w = 10\ \mu\mathrm{m}$, (c) $w = 6\ \mu\mathrm{m}$, (d) $w = 2\ \mu\mathrm{m}$, (e) $w = 0.1\ \mu\mathrm{m}$, (f) $w = 0\ \mu\mathrm{m}$). The configuration framed in red (configuration (d)) refers to the structure used in the experiments.

14. SI 14: Measurements of modal attenuation

Experimentally, the losses of a water-filled ARE fiber were determined by the cut-off method using successive shortening of the sample length and subsequent power transmission measurement (Fig. S-12). Linear fitting (in log-scale) of the measured data results in a modal loss of $\gamma_{\mathrm{exp}} \approx 0.4\ \mathrm{dB/cm}$, which agrees well with simulated data of a water-filled ARE fiber ($\gamma_{\mathrm{sim}}(w = 2\mu\mathrm{m}) = 0.4\ \mathrm{dB/cm}$). This agreement shows (i) the high accuracy of the fiber implementation procedure and (ii) that the simulated-based designs can precisely be transferred into real-world fibers.

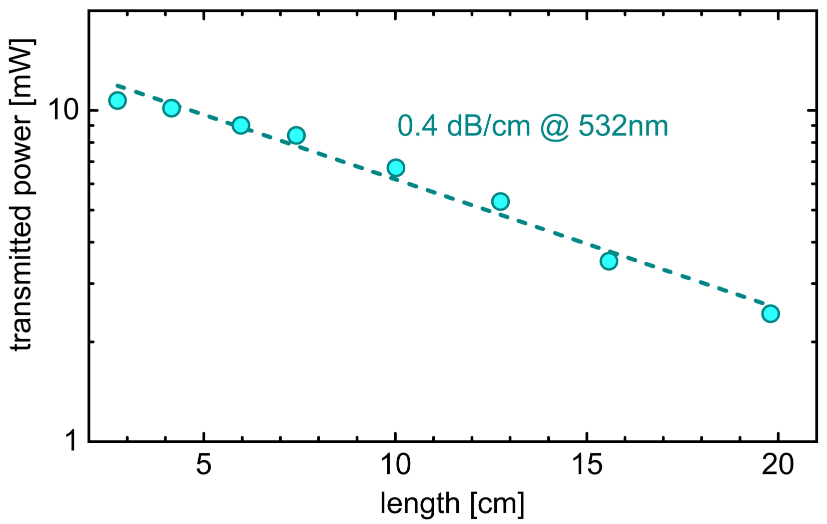

Fig. S-12: Transmitted power through the ARE as a function of sample length in case the fiber is filled with water. The dashed line corresponds to a linear fit to the data point, yielding losses of $0.4\ \mathrm{dB/\ cm}$ at the operation wavelength ($\lambda = 532\ \mathrm{nm}$).

15. SI 15: Resistance factor

An important question, which needs to be considered in the context of the presented work, is the influence of confinement on NP diffusion. The physics behind the confinement effect relies on the fact that the viscosity of the liquid can no longer be considered constant when the NP approaches the wall due to boundary effects. As a result, the viscosity increases towards the wall, which can have an influence especially in the case of small channel diameters and large NPs. Typically, this confinement-related effect is summarized in spatially dependent the resistance coefficient $R_{\mathrm{q}}(x,y)$. This parameter is commonly averaged by different models, resulting in the spatially invariant resistance factor R_s, which summarizes the confinement effect in a single quantity and ultimately defines a new viscosity ($\eta = R_{\mathrm{s}}\eta_0$, η_0: bulk viscosity). According to the existing literature, the model of H. Brenner and L. J. Gaydos [6] has been established, which has been extended to a larger parameter range by J. M. Nitsche and G. Balgi [7]. In the following, the latter model, which describes diffusion inside an infinitely extended cylinder, will be used to unravel the influence of the confinement effect on diffusion along the cylinder (i.e., fiber) axis (longitudinal direction). In all models, the crucial parameter is the ratio between the

radii of the NP and the channel $\lambda = a/R$ (a: radius of NP, R: radius of channel). This dimensionless parameter allows calculating the resistance factor independent of the actual geometric dimensions of the system investigated.

The most important parameter in the work of J. M. Nitsche und G. Balgi [7]is the diffusion correction factor k (Eq. 45 of the mentioned work) which is determined for various combinations of NP and channel radii (Fig. S-13(a)). This parameter is inverse proportional to the resistance factor ($k = 1/R_s$) and describes how the longitudinal free diffusion coefficient is changed by the confinement ($k = \widetilde{D}/D_\infty$; measured and free diffusion coefficients: $\widetilde{D}$ and D_∞). Clearly visible is a substantial deviation of the diffusion correction factor from unity, particularly in the case of small channel radii and large NPs, i.e., large values of λ. This effect can also be seen in Fig. 2 of the work of J. M. Nitsche und G. Balgi [7] (Fig. S-13(b)).

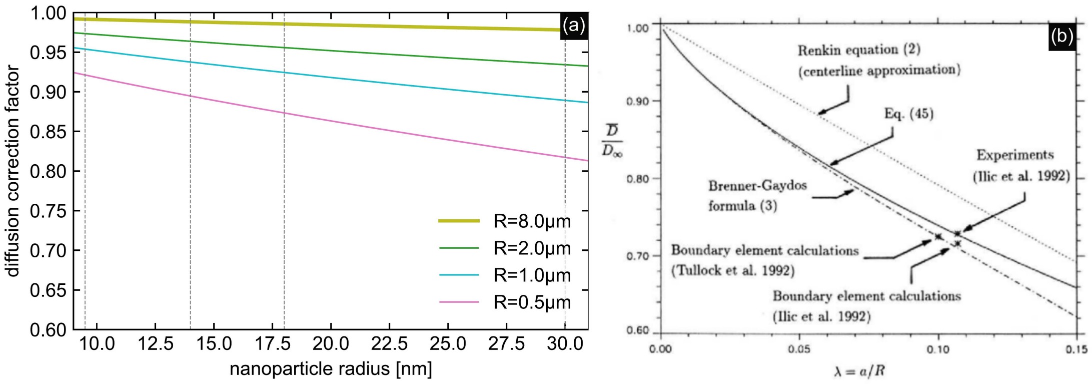

Fig. S-13: (a) Diffusion correction factor along the longitudinal direction as a function of the NP radius for four different radii of the cylindrical microchannel. The vertical dashed lines refer to the experimentally investigated NP ensembles (average ensemble hydrodynamic diameters: 9.5 nm, 14 nm, 18 nm, 30 nm). The case of $R = 8\ \mu\text{m}$ corresponding to the ARE-fiber used in the experiments presented is emphasized by a thicker line width. (b) Figure 2 of the work of J. M. Nitsche und G. Balgi [7].

To emphasize this point, example values have been extracted from the Fig. S-13(a) and are presented in Tab. S-3.

Tab. S-3: Selected value of the diffusion factor taken from Fig. S-13(a)

a [nm]	*R* [µm]	*a*/*R*	*k*
9.5	8	0.001	0.992
9.5	0.5	0.019	0.921
30	8	0.004	0.978
30	0.5	0.06	0.817

Clearly visible is that for the ARE-fiber used in this work ($R = 8\ \mu\mathrm{m}$), the impact of the confinement is small and is included into the analysis. For smaller channels, however, the diffusion coefficient indeed changes, and the resistance factor needs to be considered to obtain the correct diameter. Note that similar results are obtained through full numerical simulations of the diffusion for the transverse case (see Sec. S.11 Resistance factor correction of the Supplementary Information of Ref. [8]).

16. SI 16: Impact of confinement on MSD analysis

Another important point which has to be considered with respect to the confinement is its influence on the data analysis, i.e. the MSD analysis itself. The background is that the MSD values saturate in the case of confined diffusion and thus no linear relationship between MSD and lag times is present, making the application of the Einstein-Stokes equation questionable. Thus, for an accurate diameter determination, it is crucial that the influence of confinement is negligible for the number of lag times considered (here: $N_{\mathrm{lag}} = 2$).

To demonstrate this influence, the diffusion of NPs in a circular geometry was simulated according to the parameters used in the experiments, and the corresponding hydrodynamic diameters were calculated. For each channel diameter, 50 simulations were performed, and the results were averaged to improve statistics (Fig. S-14).

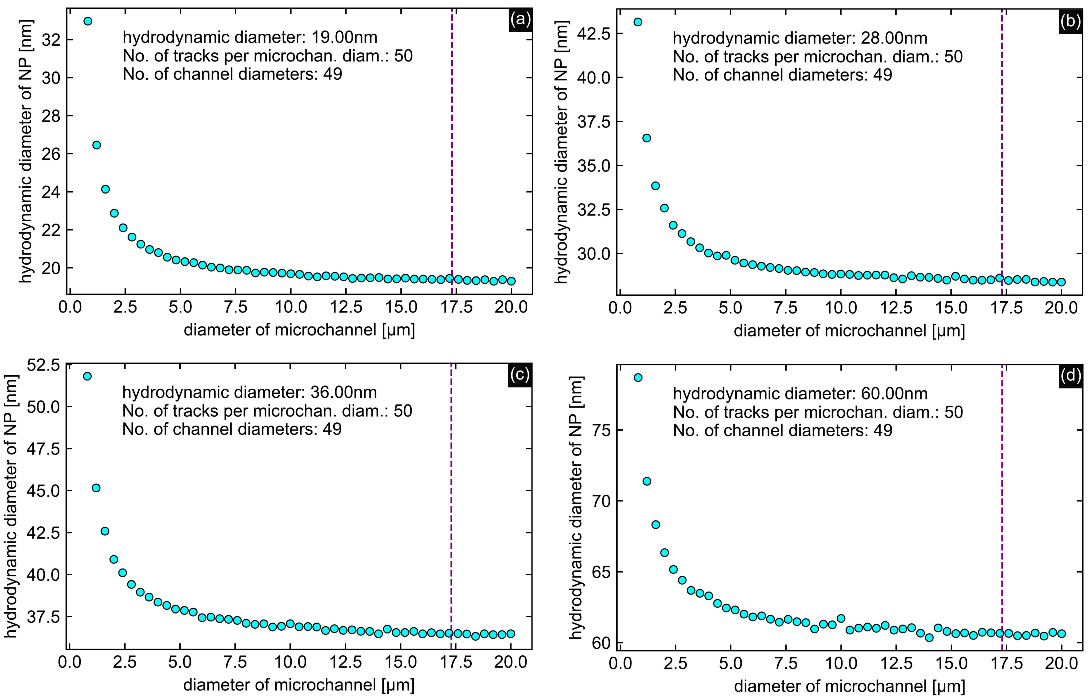

Fig. S-14: 2D nanoparticle diffusion simulations to reveal the influence of microchannel diameter on MSD analysis and thus on hydrodynamic NP diameters. Each plot shows the dependence between determined hydrodynamic NP and microchannel diameter for the four ensemble NP diameters used in the experiments ((a) 19 nm, (b) 28 nm, (c) 36 nm, (d) 60 nm). To resemble the experimental conditions as closely as possible, the parameters of the performed experiments were used in the simulations (frame rate: $\mathrm{FR} = 450\ \mathrm{fps}$, measurement time: $T = 9\ \mathrm{s}$, number of images: $N = 4050$). The increasing viscosity in the vicinity of the silica wall was neglected based on the discussion of the previous section, and a constant viscosity of water ($\eta = 1\ \mathrm{mPa \cdot s}$) was assumed. For each microchannel diameter, 50 simulations were performed, and the resulting hydrodynamic diameters were averaged. The vertical dashed purple line in each plot indicates the diameter of the used ARE-fiber ($d_\mathrm{c} = 17\ \mu m$).

The results clearly show that the channel size has a significant influence on the resulting NP diameter, particular in the case of small channel sizes. To quantify this influence, the results of two selected two microchannel diameters are compared in Tab. S-4.

Tab. S-4: Comparison of the hydrodynamic diameters at two selected microchannel and two NP diameters. The numbers are taken from Fig. S-14.

assumed hyd. NP diameter [nm]	determined hyd. diameter @ d_c=17µm [µm]	determined hyd. diameter @ d_c=0.8µm [µm]
60	60.51	78.69
36	36.47	51.81
28	28.49	43.15
19	19.41	32.97

These figures also clearly show that very small microchannel diameters impose very large errors in the resulting hydrodynamic diameter. Ultimately, this effect is a result of an insufficient frame rate ($\mathrm{FR} = 450\ \mathrm{fps}$), since for too small channel diameters saturation already impacts the values of the MSD at the first two lag times.

However, at the diameters of the ARE used in the experiments ($d_c = 17\ \mu m$), this saturation effect practically does not appear and the change in the determined diameter regarding the MSD analysis is negligible. Thus, it can be assumed that due to the sufficient frame rate ($\mathrm{FR} = 450\ \mathrm{fps}$) in the present experiments, the influence of confinement is not critical for the transverse direction.

17. SI 17: Influence of the trajectory length on the accuracy of ensemble statistics

Generally, the same number of NPs is always considered in the analysis, while by increasing $N_{\mathrm{f,min}}$, trajectories with low accuracy are increasingly omitted. As shown in [3], the standard deviation of the ensemble measurement σ_{M} is given by

$$\frac{\sigma_{\mathrm{M}}}{\overline{d_{\mathrm{M}}}} = \mathrm{CV_M} = \sqrt{\mathrm{CV_E^2} + \mathrm{CV_S^2}} \geq \sqrt{\mathrm{CV_E^2} + \frac{2}{N_{\mathrm{f,min}} - 1}\delta}$$

with the corresponding average diameter $\overline{d_{\mathrm{M}}}$ (corresponding to the symbol μ in Fig. S-3) the measured CV $\mathrm{CV_M}$ and the CVs of the NP ensemble and the system $\mathrm{CV_E}$ and $\mathrm{CV_S}$. Due to the high localization accuracy of the FaNTA approach, it can be roughly assumed that $\delta \approx 1$. If the mean NP diameter is constant for all $N_{\mathrm{f,min}}$ (as it holds in

the experiments discussed here), the standard deviation of the measurement σ_{m} depends on $N_{\mathrm{f,min}}$ according to the above equation. In the case that the CV value of the system is exclusively correlated to data analysis (i.e., the instrumental measurement error is negligibly small), $\mathrm{CV}_{\mathrm{S}}^2 = \frac{2}{N_{\mathrm{f,min}}-1}$. For this situations, Fig. S-15 shows that longer trajectories are essential to approach the CV-values of the NP ensemble, i.e., precise ensemble characterization requires long trajectories.

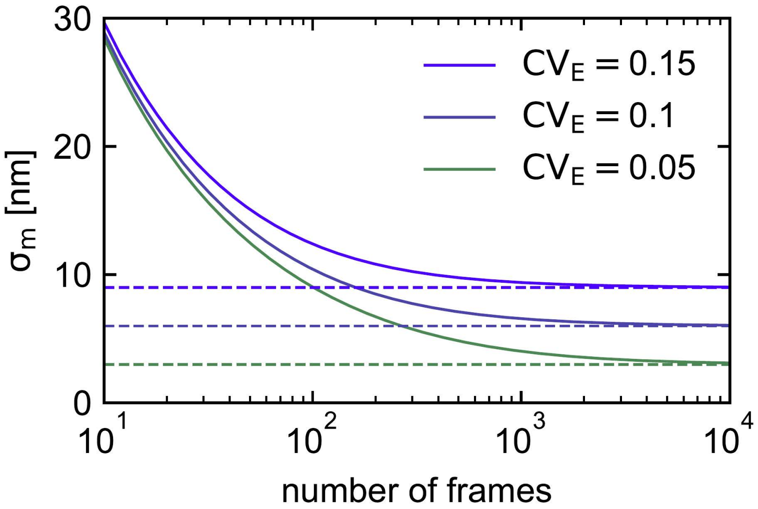

Fig. S-15: Standard deviation of the measurement as a function of the minimal number of frames for three different CV-values of the NP ensemble (defined in the legend).

This trend is also evident in Fig. 3(f), where the relationship between $N_{\mathrm{f,min}}$ and the CV value of the measurement is shown for the experimental data. It should be mentioned again that the dependency shown in Fig. S-15 is only meaningful as long as the mean diameter is almost the same for all $N_{\mathrm{f,min}}$ values considered, which holds for the present experiments.

18. SI 18: Simulation of particle diffusion for large lag times

The experiments presented in this study do not reach complete saturation within the given measurement time due to the large channel diameter. However, it is apparent that the curve deviates from its initial linear evolution towards higher lag times. To show the saturation effect, we decided here to simulate the 2D diffusion of the NPs used in the experiments for a much longer acquisition time ($T_{\mathrm{total}} = 120\ \mathrm{s}$) using two

microchannel diameters (Fig. S-16, $d_c = 4$ μm and $d_c = 17$ μm). Note that the larger channel diameter corresponds to the value of the ARE fiber used in the experiments. For the small channel diameter, the saturation of the MSD curve can be clearly seen, with, as mentioned in the manuscript, this effect starting earlier for smaller NPs due to faster diffusion. For the larger channel, this effect is much less pronounced and essentially a deviation from the linear evolution of the MSD-lag time dependence is observed.

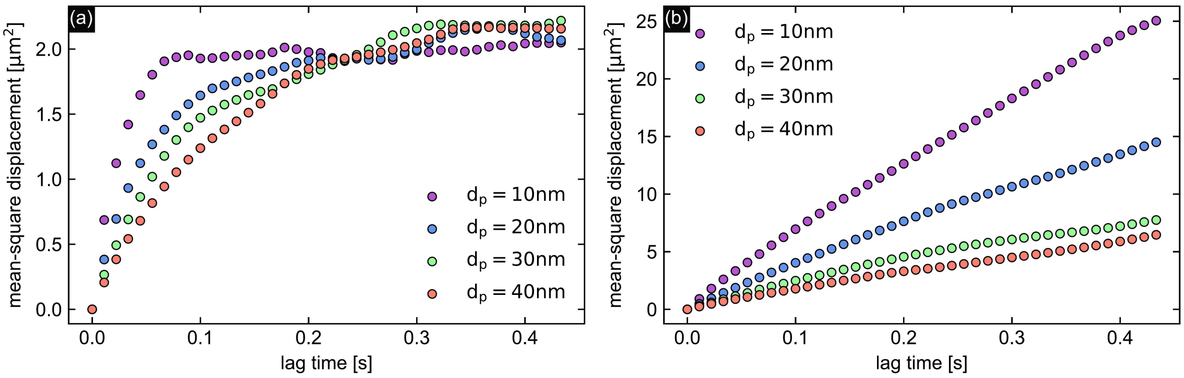

Fig. S-16: Mean square displacement (MSD) analysis of simulated data of a NP diffusing in a circular geometry for four different NPs. The two plots refer to two diameters of the disk ((a) $d_c = 4\ \mu m$, (b) $d_c = 17\ \mu m$). The analysis if performed along a selected transverse axis (here x-axis). The parameters are as follows: frame rate: $\mathrm{FR} = 450\ \mathrm{fps}$, measurement time: $T_{\mathrm{total}} = 120\ \mathrm{s}$, number of images: $N = 60000$). The increasing viscosity near the silica wall was neglected, and a constant viscosity of water ($\eta = 1\ \mathrm{mPa} \cdot s$) was assumed.

19. SI 19: Optimization of light incoupling

In the following, the light coupling procedure into the ARE is described: The optimization of the butt coupling is performed manually and includes the adjustment of the delivery fiber in xyz-direction and the rotation of the SEF around its axis. Here, the delivery fiber is mounted on a fiber launching stage (MDE122, Elliot Scientific), ensuring a sufficient adjustment distance of $2\ \mathrm{mm/axis}$ with a resolution of $20\ \mathrm{nm}$. The SEF is located on a holder which allows manual rotation of the fiber, aiming to position the SEF in such a way that the contact point of ARE and jacket faces the objective. The light at the output of the ARE, i.e., SEF is used as an indicator for the quality of the alignment regarding exciting the fundamental ARE-mode and is imaged

onto a camera chip using a lens. An example image of a measured profile of the fundamental mode is shown in Fig. S17.

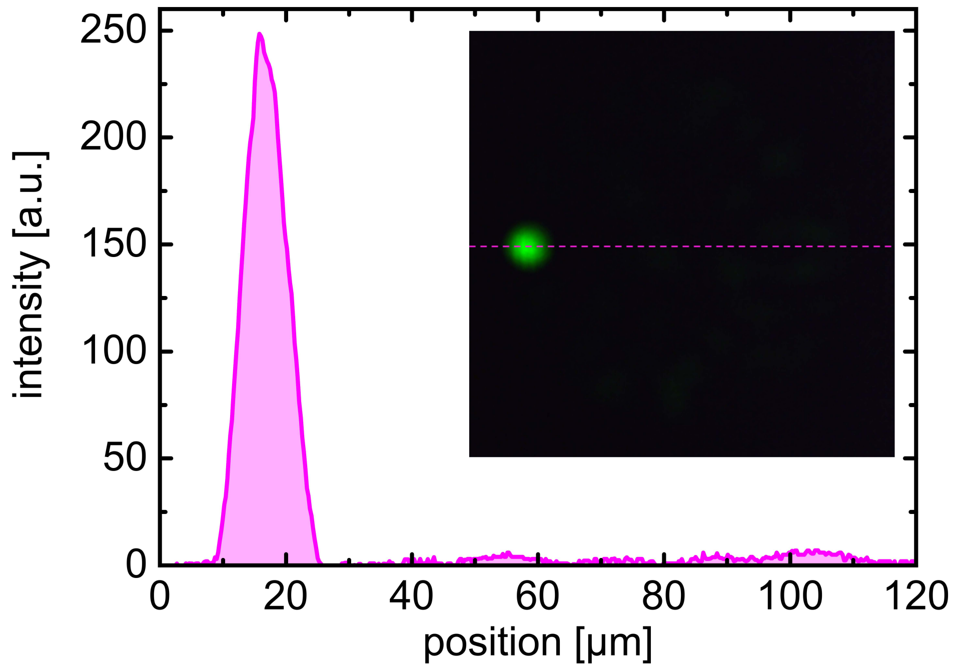

Fig. S-17: Measured intensity distribution of the fundamental mode inside the ARE along the symmetry axis of the cross-section, taken during butt coupling optimization. The inset shows the image acquired, with the dashed line indicating the axis along which the line scan is performed.

20. SI 20: Description of z-score filtering

The aim of the procedure is to sort out NPs that do not belong to the Ultra Uniform NP ensemble by means of a statistical evaluation. Here we use the so-called z-score, which describes the relationship of a value to the mean value of a group of values. If a NP has a z-score above a certain $z_{\max}$, then it is most likely an outlier (agglomerate, dirt, etc.) and must be sorted out. Note that it is important to consider that the standard deviation in the context of NTA depends on trajectory length. The details of the data evaluation procedure are as follows:

- The starting point is a matrix of the measured values with the following columns: NP-ID, diameter, diffusion coefficient, trajectory length, and brightness.
- This table is sorted with respect to trajectory length and thus accuracy.

- Then the z-score of each trajectory is determined, where the parameters individual diameter, mean diameter and individual standard deviation are necessary.
    - Individual diameter: This parameter is known
    - Mean diameter: Here the median of all diameters is used here, since the median corresponds to the mean value in a normal distribution (including outliers that are to be eliminated).
    - Individual standard deviation: This parameter is difficult to determine analytically and is therefore approximated here by taking into account objects of comparable trajectory length. For this purpose, a rolling standard deviation is used (similar to the rolling/moving/running average), which determines the standard deviation in a window of certain width around the data point of interest. This value is assigned to the data point as the standard deviation.
- As next step, the z-score of each trajectory is determined
- Then the filtering according to the $z_{\max}$ is conducted.
- If at least one NP is filtered out, the entire analysis starts over until it converges and all NP fit the hypothesis of normal distribution.

In the current analysis, we use $z_{\max} = 2.576$, which corresponds to one false event for 1000 events and 100 data points for each rolling window. If fewer trajectories are evaluated, the value is limited to the number of objects. A direct comparison of filtered and unfiltered data is shown in Fig. SI-18, revealing that statistical outliers can be effectively suppressed by this filtering procedure.

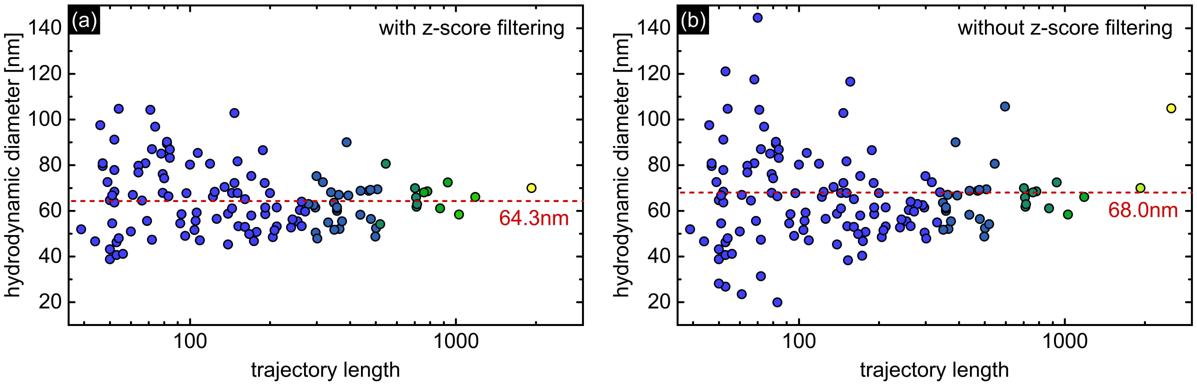

Fig. S-18: Comparison of results of MSD data analysis (a) with and (b) without z-score filtering of the same experimentally determined data set (physical diameter of gold nanoparticle $d_{\mathrm{p}} = 50\ \mathrm{nm}$.

List of mathematical symbols

symbol	parameter
<>	expectation value operator
A_{ch}	area of ARE
b	scattering coefficient of liquid
c	concentration
CV	coefficient of variation
CV_E	coefficient of variation of the ensemble
CV_M	coefficient of variation of the ensemble measurement
CV_S	coefficient of variation of the system
d_M	average diameter of the ensemble measurement
D	diffusion coefficient
$\widetilde{D}$	diffusion coefficients (measured)
D_∞	diffusion coefficients (free)
d_c	ARE diameter
$d_{DLS,\ a}$	hydrodynamic diameter of NP (DLS measurement; authors)
$d_{DLS,\ m}$	hydrodynamic diameter of NP (DLS measurement; manufacturer)
d_h	hydrodynamic diameter of NP
$\overline{d_M}$	average diameter of NP
$d_{FaNTA\text{-}long}$	hydrodynamic diameter of NP (Fa.NTA measurement, longitudinal direction)
$d_{FaNTA\text{-}trans}$	hydrodynamic diameter of NP (Fa.NTA measurement, transversal direction)
d_p	physical diameter of NP
$d_{TEM,\ M}$	diameter of NP (transmission electron microscopy measurement; manufacturer)
FR	frame rate
i	lag frame
j	frame number
k	diffusion correction factor
l	observation length of fiber
MSD	mean square displacement
n	refractive index
NA	numerical aperture
N	number of images
N_f	trajectory length
$N_{f,min}$	minimum trajectory length
N_{lag}	number of lag times
N_p	number of evaluated particles, parallel tracked particles
P	power
P_{ave}	average power

P_n	normalized power
R	Rayleigh ratio
R_g	resistance coefficient
R_s	resistance factor
$P_{max}^{(heat)}$	power limit to prevent heating of AuNp
T	temperature
T	total periode
T	total measurement time
t	time
t_m	time of each measurement
v	drift velocity
V	volume
w	wall thickness of anti-resonant ring
x	transversal direction (within fiber cross-section)
z	axial direction (perp. to fiber cross-section)
δ	localization error correction factor
Δx	misplacement along x
Δx_{brown}	brownian motion
Δx_{drift}	drift induced displacement in *x* direction
Δt	lag time
ΔT_p	reduction of power transmission
ε	rel. error
γ_{sim}	loss (simulation)
γ_{exp}	loss (experiment)
μ	mean value
η	dynamic viscosity
η_0	dynamic bulk viscosity
λ	wavelength
σ	standard deviation
σ^2	localization accuracy
σ_{abs}^2	absorption cross-section of NP
σ_d	statistical error of MSD analysis
σ_M	standard deviation of the ensemble measurement
σ_P	standard deviation of transmitted power
σ_{scat}^2	scattering cross-section of NP
σ_{ext}^2	extinction cross-section of NP
τ	exposure time

21. Supplementary References

1. Michalet, X. & Berglund, A. J. Optimal diffusion coefficient estimation in single-particle tracking. *Phys. Rev. E* **85**, (2012).
2. Dechadilok, P. & Deen, W. M. Hindrance factors for diffusion and convection in pores. *Ind. Eng. Chem. Res.* **45**, 6953–6959 (2006).
3. Nissen, M. *et al.* Nanoparticle Tracking in Single-Antiresonant-Element Fiber for High-Precision Size Distribution Analysis of Mono- and Polydisperse Samples. *Small* **18**, (2022).
4. Parfitt, G. D. & Wood, J. A. Light scattering from binary mixtures of water, methanol and ethanol. *Trans. Faraday Soc.* **64**, 2081-2090 (1968)
5. Coe, J. R. & Godfrey, T. B. Viscosity of water. *Journal of Applied Physics* **15**, 625–626 (1944).
6. Brenner, H. & Gaydos, L. J. The constrained brownian movement of spherical particles in cylindrical pores of comparable radius: Models of the diffusive and convective transport of solute molecules in membranes and porous media. *Journal of Colloid and Interface Science* **58,** 312–356 (1977).
7. Nitsche, J. M. & Balgi, G. Hindered Brownian Diffusion of Spherical Solutes within Circular Cylindrical Pores. *Ind. Eng. Chem. Res.* **33**, 2242–2247 (1994).
8. Jiang, S., Förster, R., Lorenz, A. & Schmidt, M. A. Three-dimensional tracking of nanoparticles by dual-color position retrieval in a double-core microstructured optical fiber. *Lab Chip* **21**, 4437–4444 (2021).
