# Supplementary material for: Characterization of diffusing sub-10 nm nano-objects using single anti-resonant element optical fibers
Source: Nat Commun. 2023 Jun 5;14:3247. doi: 10.1038/s41467-023-39021-3 (PMC10241938; doi:10.1038/s41467-023-39021-3)
Supplement: Supplementary file 3 — Lasing Reporting Summary [file 41467_2023_39021_MOESM3_ESM.pdf]

naturereseach

Corresponding author(s): Markus A. Schmidt

Lasing Reporting Summary

Nature Research wishes to improve the reproducibility of the work that we publish. This form is intended for publication with all accepted papers reporting claims of lasing and provides structure for consistency and transparency in reporting. Some list items might not apply to an individual manuscript, but all fields must be completed for clarity.

For further information on Nature Research policies, including our data availability policy, see Authors & Referees.

ü Experimental design

Please check: are the following details reported in the manuscript?

1. Threshold

Plots of device output power versus pump power over a wide range of values indicating a clear threshold

[ ] Yes
[x] No

out of the scope of this study

2. Linewidth narrowing

Plots of spectral power density for the emission at pump powers below, around, and above the lasing threshold, indicating a clear linewidth narrowing at threshold

[ ] Yes
[x] No

out of the scope of this study

Resolution of the spectrometer used to make spectral measurements

[ ] Yes
[x] No

out of the scope of this study

3. Coherent emission

Measurements of the coherence and/or polarization of the emission

[ ] Yes
[x] No

out of the scope of this study

4. Beam spatial profile

Image and/or measurement of the spatial shape and profile of the emission, showing a well-defined beam above threshold

[x] Yes
[ ] No

supplementary information, SI, Sec. SI19,Fig. S-17; main text, Fig. 2d.

5. Operating conditions

Description of the laser and pumping conditions
Continuous-wave, pulsed, temperature of operation

[x] Yes
[ ] No

cw

Threshold values provided as density values (e.g. W cm^{-2} or J cm^{-2}) taking into account the area of the device

[ ] Yes
[x] No

not relevant for this study

6. Alternative explanations

Reasoning as to why alternative explanations have been ruled out as responsible for the emission characteristics
e.g. amplified spontaneous, directional scattering; modification of fluorescence spectrum by the cavity

[ ] Yes
[x] No

not relevant for this study, laser is not target of the investigation

7. Theoretical analysis

Theoretical analysis that ensures that the experimental values measured are realistic and reasonable
e.g. laser threshold, linewidth, cavity gain-loss, efficiency

[ ] Yes
[x] No

not relevant for this study, laser is not target of the investigation

8. Statistics

Number of devices fabricated and tested

[ ] Yes
[x] No

not relevant for this study, laser is not target of the investigation

Statistical analysis of the device performance and lifetime (time to failure)

[ ] Yes
[x] No

not relevant for this study, laser is not target of the investigation
